# The structure of TRAF7 coiled-coil trimer provides insight into its function in zebrafish embryonic development

**DOI:** 10.1093/jmcb/mjad083

**Published:** 2024-01-04

**Authors:** Xiaozhen Song, Ruixing Hu, Yi Chen, Man Xiao, Hong Zhang, Shengnan Wu, Qing Lu

**Affiliations:** Molecular Diagnostic Laboratory, Shanghai Children's Hospital, School of Medicine, Shanghai Jiao Tong University, Shanghai 200040, China; Bio-X Institutes, Key Laboratory for the Genetics of Developmental and Neuropsychiatric Disorders (Ministry of Education), Shanghai Jiao Tong University, Shanghai 200030, China; Laboratory of Development and Diseases and State Key Laboratory for Medical Genomics, Shanghai Institute of Hematology, Ruijin Hospital, Shanghai Jiao Tong University School of Medicine, Shanghai 200025, China; Molecular Diagnostic Laboratory, Shanghai Children's Hospital, School of Medicine, Shanghai Jiao Tong University, Shanghai 200040, China; Molecular Diagnostic Laboratory, Shanghai Children's Hospital, School of Medicine, Shanghai Jiao Tong University, Shanghai 200040, China; Molecular Diagnostic Laboratory, Shanghai Children's Hospital, School of Medicine, Shanghai Jiao Tong University, Shanghai 200040, China; Bio-X Institutes, Key Laboratory for the Genetics of Developmental and Neuropsychiatric Disorders (Ministry of Education), Shanghai Jiao Tong University, Shanghai 200030, China

**Keywords:** TRAF7, coiled-coil domain, crystal structure, trimerization, embryonic development

## Abstract

TRAF7 serves as a crucial intracellular adaptor and E3 ubiquitin ligase involved in signal transduction pathways, contributing to immune responses, tumor progression, and embryonic development. Somatic mutations within the coiled-coil (CC) domain and WD40 repeat domain of TRAF7 could cause brain tumors, while germline pathogenic mutations contribute to severe developmental abnormalities. However, the precise molecular mechanism underlying TRAF7 involvement in embryonic development remains unclear. In this study, we employed zebrafish as an *in vivo* model system. TRAF7 knock down caused defects in zebrafish embryonic development. We determined the crystal structure of TRAF7 CC domain at 3.3 Å resolution and found that the CC region trimerization was essential for TRAF7 functionality during zebrafish embryonic development. Additionally, disease-causing mutations in TRAF7 CC region could impair the trimer formation, consequently impacting early embryonic development of zebrafish. Therefore, our study sheds light on the molecular mechanism of TRAF7 CC trimer formation and its pivotal role in embryonic development.

## Introduction

The tumor necrosis factor receptor-associated factor (TRAF) family consists of seven proteins (TRAF1–7) in mammals that directly or indirectly interact with tumor necrosis factor receptor (TNFR) proteins, thereby mediating activated TNFR signaling (Bouwmeester et al., 2004; Xu et al., 2004; Ha et al., 2009). TRAF family proteins are involved in various biological functions, including morphogenesis, apoptosis, stress response, and immune response (Zotti et al., 2012). TRAF7, the most recently identified member of the TRAF family, has gained attention due to its involvement in pathogenic processes (Bouwmeester et al., 2004). Somatic mutations in *TRAF7* are implicated in tumorigenesis (Zotti et al., 2017), most reported for meningioma and mesothelioma. Multiple germline mutations in *TRAF7* have been reported in >50 individuals with developmental disorders mainly featuring cardiac, facial, and digital anomalies with developmental delay, suggesting a critical role for TRAF7 in early development (Tokita et al., 2018; Castilla-Vallmanya et al., 2020; Paprocka et al., 2021). These mutations are notably enriched within the coiled-coil (CC) domain and WD40 repeat domain (Zotti et al., 2017; Castilla-Vallmanya et al., 2020).

TRAF family proteins exhibit a common domain organization, including an N-terminal RING domain, one or several zinc finger (ZF) domains (excluding TRAF1), a CC domain (known as the TRAF-N domain), and a TRAF-C domain (excluding TRAF7) (Rothe et al., 1994). TRAF7 is a noncanonical member of the TRAF family, with a long CC domain and distinct WD40 repeats instead of the TRAF-C domain. The RING domain of TRAF7 has E3 ubiquitin ligase activity akin to that of other TRAF family members. Mitogen-activated protein/extracellular signal-regulated kinase (MAP/ERK) kinase kinase 3 (MEKK3) can facilitate TRAF7 phosphorylation and autoubiquitination to stabilize TRAF7 (Bouwmeester et al., 2004; Xu et al., 2004). TRAF7 also mediates ubiquitination or SUMOylation of several proteins (Morita et al., 2005; Tsikitis et al., 2010).

Previous studies showed that TRAF1–6 can form homotrimers or heterotrimers mediated by the CC domain (Rothe et al., 1994; Park et al., 1999, 2018; Zapata et al., 2001). Coiled coils, found in ∼3% of proteins, exhibit significant binding capacity. Initially described by Crick (1953) and Pauling and Corey (1953), researches on coiled coils have focused on their variable polymerization and versatile target recognition. The canonical coiled coils show a seven-residue sequence periodicity, with hydrophobic residues typically occupying the first and fourth positions of the heptad repeat to form knob-into-hole packing interactions (Lupas and Bassler, 2017). For instance, in the context of TRAF1/2, both proteins can form homotrimers (Park et al., 1999; Kim et al., 2016), and they preferentially form a TRAF1:(TRAF2)_2_ heterotrimer, displaying enhanced interaction with cIAP2 compared to TRAF2 alone (Zheng et al., 2010). However, whether TRAF7 can form a homotrimer via its CC domain has yet to be characterized.

To uncover the function and molecular mechanism of TRAF7 in early embryonic development, we employed zebrafish as an *in vivo* model system and conducted structural and biochemical characterizations of TRAF7. Meanwhile, we characterized two significant mutations (K346E and R371G) in the CC region, known to cause developmental disorders. This investigation sheds light on the pathogenicity of TRAF7 mutations.

## Results

### Spatiotemporal expression of traf7 during early development of zebrafish

**Figure 1:**
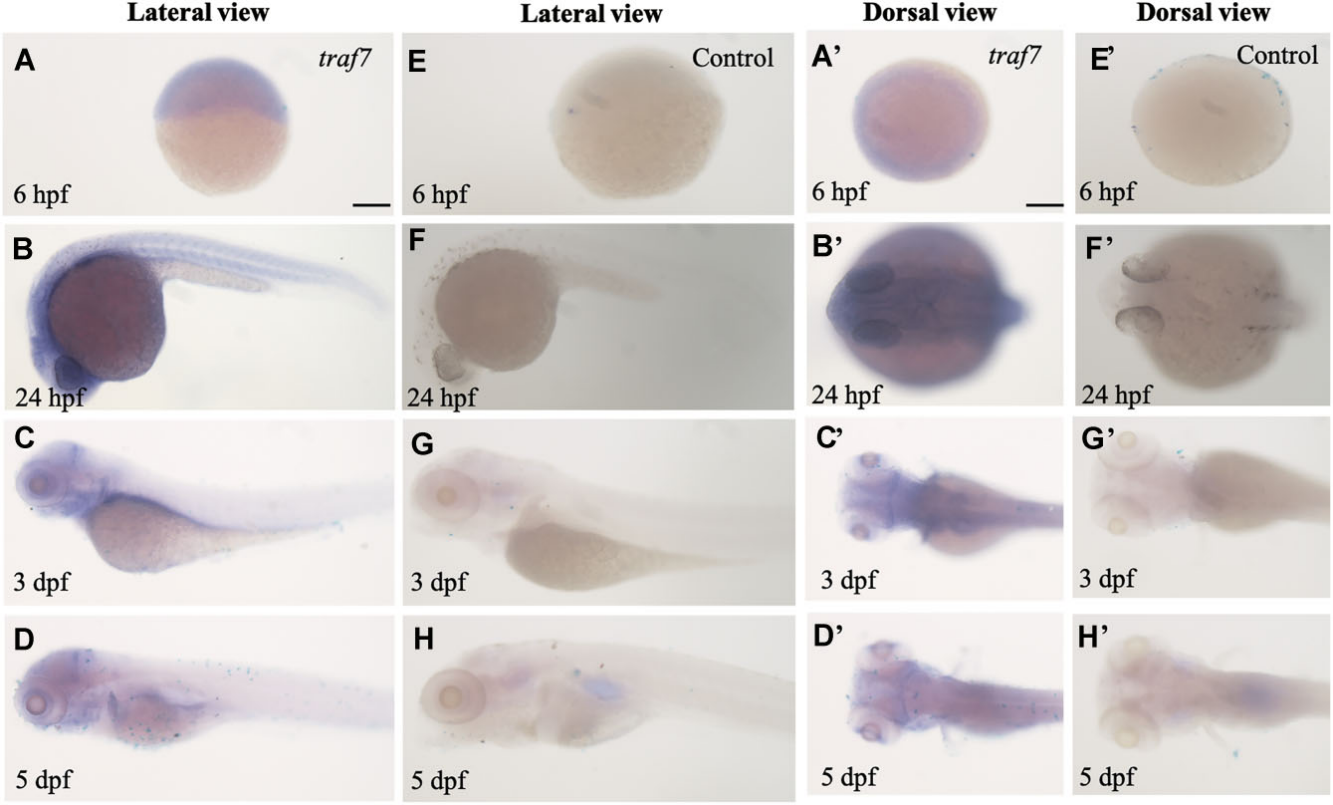
Spatiotemporal expression of *traf7* in zebrafish development. (**A**–**D** and **A**'–**D**') WISH staining delineates zebrafish *traf7* expression in the brain during early embryonic development. Lateral view (**A**–**D**) and dorsal view (**A**'–**D**') of the zebrafish larvae at different stages. Scale bar, 200 μm. (**E**–**H** and **E**'–**H**') The control group without *traf7*-specific riboprobe.

Given the high conservation of TRAF7 sequences among vertebrates (Figure 3E), the zebrafish system presents a promising model to study TRAF7 function during embryogenesis. Using specific antisense riboprobes (Supplementary Table S1), we performed whole-mount *in situ* hybridization (WISH) to examine the expression dynamics of *traf7* at different zebrafish developmental stages, including 6 hours post-fertilization (hpf), 1 day post-fertilization (dpf), 3 dpf, and 5 dpf (Figure 1A–D and A'–D'). A control group without *traf7*-specific riboprobes was included for comparison (Figure 1E–H and E'–H'). Notably, *traf7* mRNA was detectable at 6 hpf, the shield stage (Figure 1A and A'). By 1 dpf, *traf7* expression started to show tissue specificity. The staining of *traf7* was concentrated around the yolk sac (Figure 1B), especially at the height of the head (Figure 1B'). By 3–5 dpf, *traf7* expression became restricted in the head, especially at the midbrain–hindbrain boundary (Figure 1C, C', D, and D'). These results demonstrated the spatial and temporal expression patterns of *traf7* during early embryonic development of zebrafish. The detection of zebrafish *traf7* expression before segmentation and the sustained expression in the brain underscores the significance of *traf7* in early developmental processes.

### Traf7 knock down affects zebrafish embryonic development

To explore the role of endogenous *traf7* in zebrafish embryonic development, we designed three antisense morpholino oligonucleotides (MOs), *traf7* ATG-MO against the ATG start codon as well as *traf7* E3i3-MO and *traf7* E4i4-MO, to prevent proper splicing (Supplementary Table S2), to knock down zebrafish Traf7.

**Figure 2:**
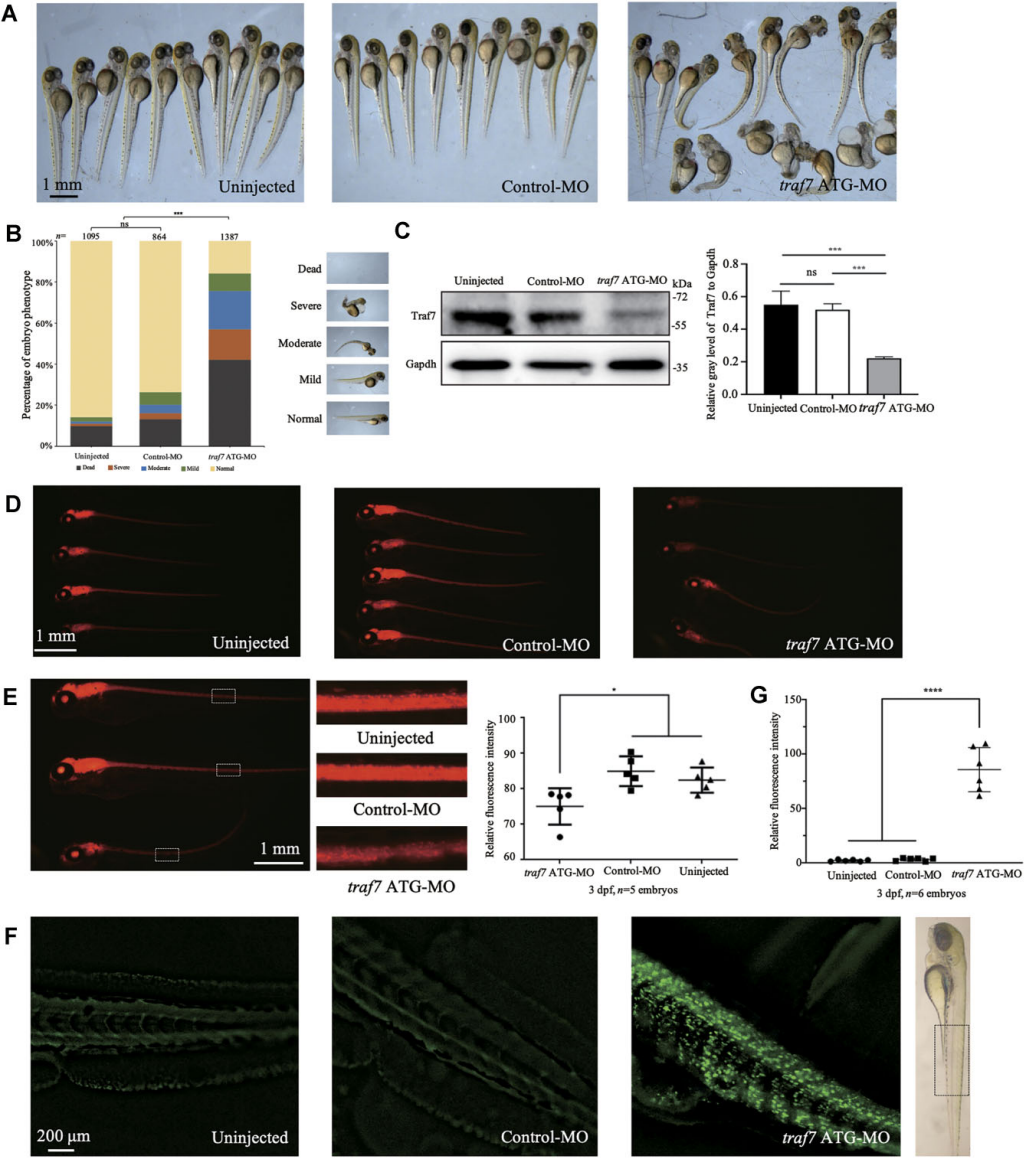
Knockdown of Traf7 causes developmental defects in zebrafish. (**A**) Gross morphology of zebrafish embryos after *traf7* ATG-MO injection. The images were taken at 3 dpf. The morphology of the uninjected and control-MO groups appear normal, whereas the embryos injected with *traf7* ATG-MO show body defects, including unconsumed yolk sac, microcephaly, curved body axis, pericardial edema, and short body. (**B**) The embryos were divided into five categories according to the severity of the body defects (normal, mild, moderate, severe, and dead). (**C**) Injection of *traf7* ATG-MO into embryos reduces Traf7 protein levels (***P < 0.01). (**D** and **E**) Knockdown of Traf7 affects neuronal development in the Tg(*huC*:*RFP*) zebrafish line, as indicated by the reduced fluorescence intensity (*n* = 5 embryos per group, *P < 0.05). (**F** and **G**) Knockdown of Traf7 induces neural cell death. The intense fluorescent apoptotic foci in the spinal cord were detected by the TUNEL assay at 3 dpf (*n* = 6 embryos per group, ****P < 0.0001).

The wild-type strain zebrafish were reared under standard laboratory conditions. Reverse transcription‒polymerase chain reaction (PCR) showed that *traf7* E3i3-MO and *traf7* E4i4-MO effectively reduced *traf7* expression compared with control-MO (Supplementary Figure S1B and Table S1). Knockdown of Traf7 by *traf7* ATG-MO, *traf7* E3i3-MO, and *traf7* E4i4-MO led to severe developmental delay and body defects in zebrafish embryos, including unconsumed yolk sac, microcephaly, obvious curved body axis, pericardial edema, and short body (Supplementary Figure S1A), compared to the uninjected and control-MO groups. Because the *traf7* ATG-MO group showed a better deformity ratio and stabler dose-dependent effect per injection, we used *traf7* ATG-MO (2 ng) to knock down Traf7 in the following experiments (Supplementary Figure S2). Injection of *traf7* ATG-MO into embryos resulted in ∼60% reduction in Traf7 protein expression (Figure 2C) (anti-TRAF7, Proteintech) and led to a phenotype comprising microcephaly, obvious curved body axis, and short body in ∼80% of living zebrafish at 3 dpf (Figure 2A and B).

According to the severity of morphology, the injected zebrafish embryos were clustered into five categories (Figure 2B). It is worth noting that 2 ng *traf7* ATG-MO caused ∼40% mortality at 3 dpf, while the same dose of control-MO caused a much lower mortality rate (∼12%), not significantly different from that of the uninjected group (∼10%) (Figure 2B). The proportion of zebrafish with obvious defects in the control-MO group did not significantly differ from that in the uninjected group (Figure 2B).

The defects observed in *traf7* MO-injected zebrafish, such as microcephaly and curly spinal cord, also suggest a role for *traf7* in zebrafish nervous system development. To visualize the effects of Traf7 knock down on zebrafish neurons, we employed the transgenic line Tg(*huC*:*RFP*), in which the promoter of the zebrafish neuronal marker *huC* gene is used to drive the expression of red fluorescent protein (RFP) in various neural areas. Similar to clinically reported human patients with TRAF7 variants presenting with neurodevelopmental disorders, Tg(*huC*:*RFP*) zebrafish injected with *traf7* ATG-MO showed significantly reduced *huC* fluorescent signals compared to the uninjected and control-MO groups (Figure 2D and E), suggesting fewer neurons projecting or excessive neural cell death during zebrafish development. We further performed the terminal deoxynucleotidyl transferase-mediated dUTP nick-end labeling (TUNEL) assay to examine apoptosis in 3 dpf embryos. Intense green fluorescent apoptotic foci in the spinal cord were detected in the *traf7* ATG-MO group (Figure 2F and G), suggesting prominent cell death resulting from Traf7 knock down.

### TRAF7 CC domain forms a parallel trimer

**Figure 3:**
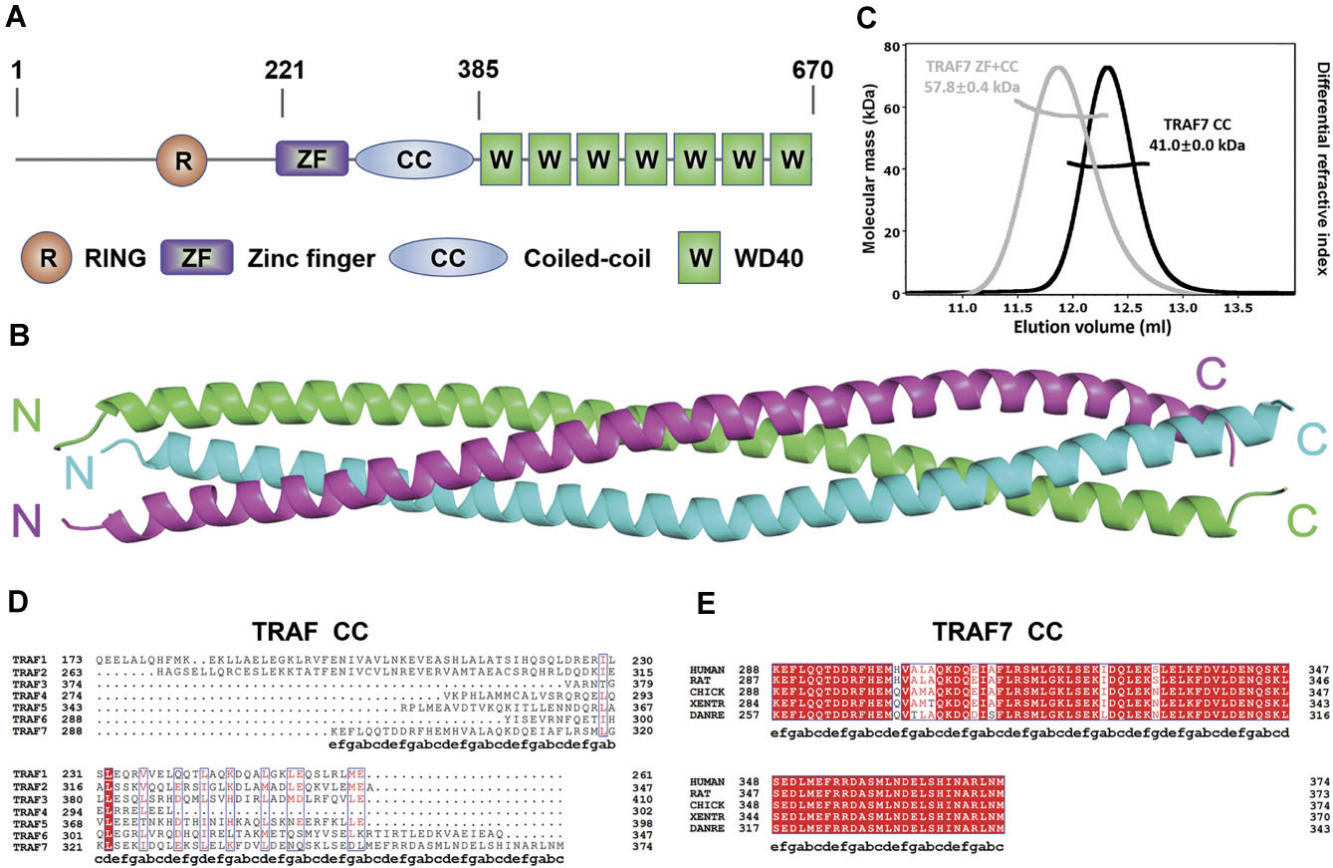
Overall structure of TRAF7 CC region. (**A**) Schematic diagram showing the domain organization of TRAF7: a RING domain, a ZF domain, a CC domain, and seven WD40 repeats. (**B**) Ribbon diagram representation of the TRAF7 CC crystal. The crystal was obtained using TRAF7 fragment 270–379 aa, and prominent electronic cloud density was observed in residues E285–L377. TRAF7 CC forms a parallel trimer, presented here with three chains in green, magenta, and cyan. (**C**) MALS measurement of TRAF7 CC (fragment 270–379 aa) and TRAF7 ZF-CC (fragment 221–379 aa) showing that the measured molecular weights align with trimeric TRAF7 in solution. Theoretical molecular weights of the trimers: TRAF7 CC, 42.3 kDa; TRAF7 ZF-CC, 59.1 kDa. (**D**) Sequence alignment of human TRAF CC regions across paralogs, revealing low sequence similarity between human TRAF7 CC and human TRAF1–6 CC. The letters a–g below the TRAF7 sequence are the residue positions of the heptad repeats in the CC region. (**E**) Sequence alignment of TRAF7 CC regions across species, demonstrating high sequence identity in human, rat, chicken, frog, and zebrafish.

Next, we set out to determine the structural composition of TRAF7. TRAF7 contains a RING domain, a ZF domain, a CC domain, and seven WD40 repeats (Figure 3A). As predicted by the MultiCoil algorithm, human TRAF7 CC has the capability to form a trimer (Supplementary Figure S3). We tested various fragments of TRAF7, and following multiple rounds of optimization, we managed to obtain well-formed crystals of human TRAF7 CC region. The structure of TRAF7 CC (fragment 270–379 aa) was solved at 3.3 Å resolution by X-ray diffraction (Figure 3B and Table 1). As predicted, TRAF7 CC forms a parallel CC trimer in the crystal, and residues E285–L377 exhibit distinct electronic cloud density and form coiled coil.

**Table 1:** **Crystal data collection and refinement statistics (PDB ID: 8IMS)**.

	TRAF7
Wavelength	0.978530 Å
Resolution range	45.09–3.304 (3.422–3.304)
Space group	P 64
Unit cell	169.923 169.923 57.055 90 90 120
Unique reflections	14379 (1393)
Multiplicity	4.07
Completeness (%)	99.75 (98.51)
Mean I/sigma(I)	8.22 (1.48)
Wilson B-factor	97.36
CC1/2	0.996 (0.527)
Reflections used in refinement	14377 (1393)
Reflections used for R-free	761 (81)
R-work	0.2600 (0.3688)
R-free	0.2733 (0.4266)
Number of non-hydrogen atoms	2250
Macromolecules	2241
Ligands	0
Solvent	9
Protein residues	277
RMS(bonds)	0.004
RMS(angles)	0.75
Ramachandran favored (%)	98.89
Ramachandran allowed (%)	1.11
Ramachandran outliers (%)	0.00
Rotamer outliers (%)	0.00
Clashscore	8.77
Average B-factor	105.07
Macromolecules	105.22
Solvent	66.38

Statistics for the highest-resolution shell are shown in parentheses.

To validate whether TRAF7 CC trimer represents the oligomerization state of the full-length TRAF7 protein, we characterized the fragments of TRAF7 with various lengths. Using the multi-angle light scattering (MALS) assay, we demonstrated that both TRAF7 CC and TRAF7 ZF-CC (fragment 221–379 aa) independently form trimers in solution (Figure 3C). Despite a relatively low sequence analogy between TRAF7 CC and that of other TRAF family proteins (Figure 3D), TRAF7 CC sequence is well conserved across diverse vertebrate species, from zebrafish (*Danio rerio*) to humans, underscoring its unique functional role (Figure 3E).

### Atomic structure of TRAF7 CC trimer

We then explored the detailed mechanism of trimerization at the atomic level. The comparison of CC domain structures of TRAF family members, which were collected from the Protein Data Bank archive (PDB) or computationally generated by AlphaFold (Kim and Park, 2020), revealed that TRAF7 has a long CC region similar to TRAF1/2 CC region (Supplementary Figure S4), indicating the potential for occupying a significant space and engaging in interactions with other proteins.

**Figure 4:**
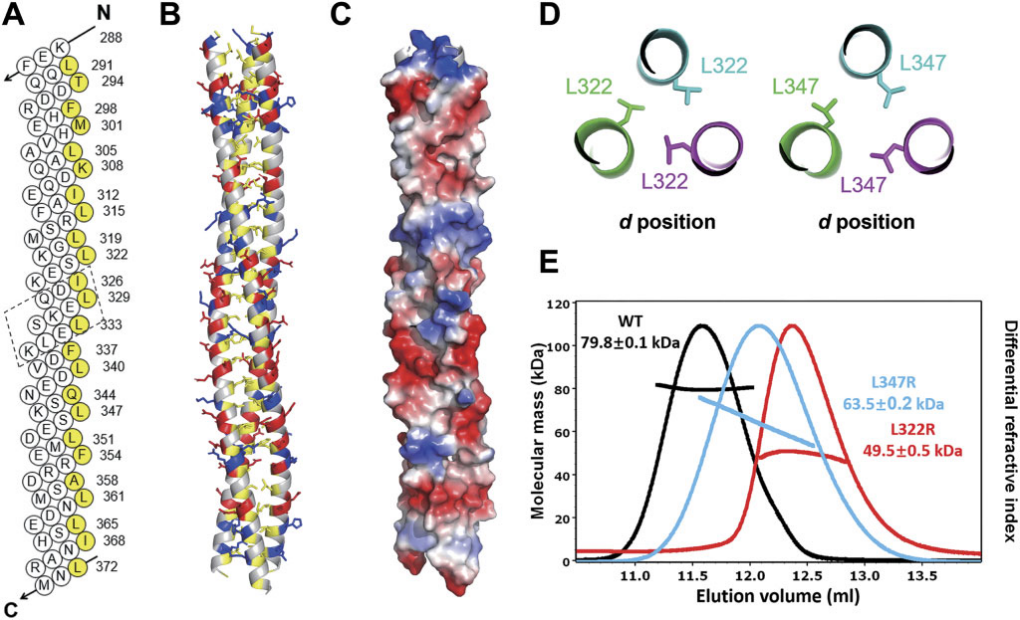
Structure validation and analysis of TRAF7 CC region. (**A**) Helical net diagram of one helical strand of TRAF7 CC trimer. One hendecad repeat is highlighted in the dashed box. The residues in the *a*/*d* positions of the heptad repeats and those in the *a*/*d*/*x* positions of the hendecad repeat are marked in yellow. (**B**) Combined ribbon and stick-ball models of TRAF7 CC to show the trimer interfaces. The main chains are shown as ribbons, and the side chains of hydrophobic and charged residues are shown as tick balls. (**C**) The surface charge distribution of TRAF7 CC. Many charged residues with large side chains are distributed on the trimer surface to protect the hydrophobic core. (**D**) Close-up view of L322 and L347 in the trimer interfaces. These hydrophobic residues are at the *d* positions, vital to form the trimeric core. (**E**) MALS measurement of TRAF7 CC molecular weights. A comparison with the wild-type TRAF7 CC (WT) shows lower molecular masses for both L322R and L347R mutants, indicating a weakening of the trimeric structure in solution. TRAF7 CC proteins (WT and mutant) were TrxA-tagged at the N-terminus for this experiment. The theoretical molecular weight of the WT trimer is 84.9 kDa.

Detailed structural analysis revealed that TRAF7 CC comprises 10 regular heptad repeats and one hendecad repeat interspersed between the heptad repeats (Figure 4A). The helical diagram analysis revealed that the residues at the *a* and *d* positions of the heptad/hendecad repeats of TRAF7 CC (residues in yellow in Figure 4A) are predominantly hydrophobic residues, which are mainly responsible for forming the folding core of the trimer (Figure 4B). Charge residues in the *e* and *g* positions further stabilize the CC structure by shielding the hydrophobic core from the surrounding solution (Figure 4B and C).

To further illustrate the significance of the hydrophobic core, we designed a monomeric TRAF7 mutant protein, introducing single-point substitutions at critical hydrophobic sites (Figure 4D). MALS assays showed that the L322R and L347R mutants displayed significantly lower molecular weights than the wild-type TRAF7 CC (Figure 4E). This observation emphasized that these mutants lacked the tertiary structure present in the wild-type protein, further highlighting the pivotal role of the hydrophobic core.

### TRAF7 CC is involved in human diseases

**Figure 5:**
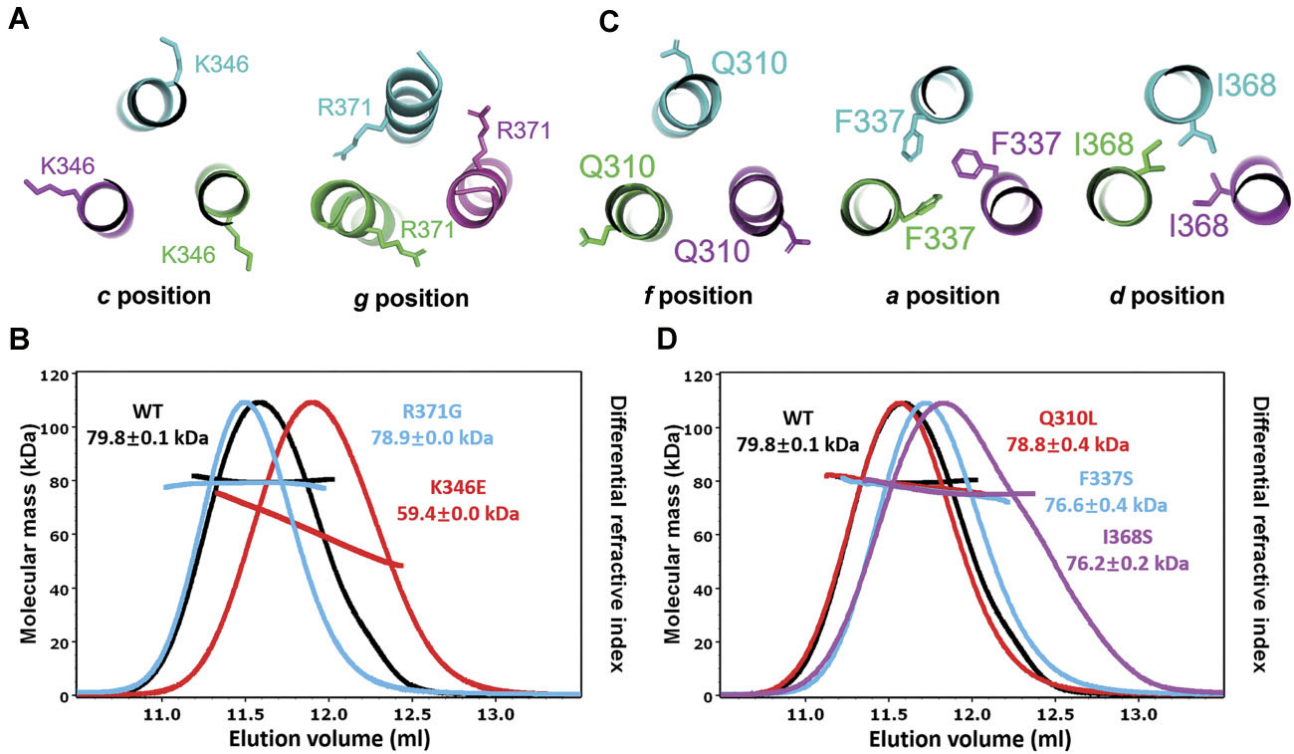
TRAF7 CC is involved in human diseases. (**A**) Close-up view of K346 and R371 in the trimer interfaces. K346 is at the *c* position, and R371 is at the *g* position. (**B**) MALS measurement of molecular weights of wild-type TRAF7 CC (WT) and the K346E and R371G mutants. (**C**) Close-up view of Q310L, F337S, and I368S in the trimer interfaces. Q310 is at the *f* position, F337 is at the *a* position, and I368 is at the *d* position. (**D**) MALS measurement of TRAF7 CC molecular weights of WT and the Q310L, F337S, and I368S mutants. The elution peaks of F337S and I368S are shifted to the right.

To elucidate the functional role of TRAF7 CC region, we analyzed the molecular properties of several TRAF7 variants involved in human diseases. Recent findings have identified *TRAF7* mutations in multiple cases exhibiting similar developmental disorder symptoms. Among these, K346E and R371G mutations, as reported by Tokita et al. (2018), are located in TRAF7 CC region (Figure 5A). MALS assays revealed that K346E exhibited a lower molecular weight compared to the wild-type TRAF7 CC, while R371G did not present a significant difference in molecular weight (Figure 5B), suggesting a weakened stability of the TRAF7 homotrimer caused by the K346E mutation. Hence, K346E likely influences TRAF7 function to contribute to the observed developmental disorders, while the R371G mutation may affect interactions between TRAF7 and other proteins, thereby contributing to disease pathogenesis.

In addition to developmental disorders, TRAF7 somatic variants are also involved in multiple tumors, mainly meningioma and mesothelioma. We thus analyzed three specific mutations (Q310L, F337S, and I368S) associated with tumor development by the MALS assay (Figure 5C). The results showed that the Q310L mutant behaved similar to the wild-type TRAF7 CC, while the elution peaks of the F337S and I368S variants were shifted to the right, suggesting weaker interactions between the chains of the trimer (Figure 5D), indicating that the F337S and I368S variants likely form the trimer with lower structural stability, ultimately affecting TRAF7 function.

### TRAF7 CC domain plays a crucial role in the development of zebrafish embryos

**Figure 6:**
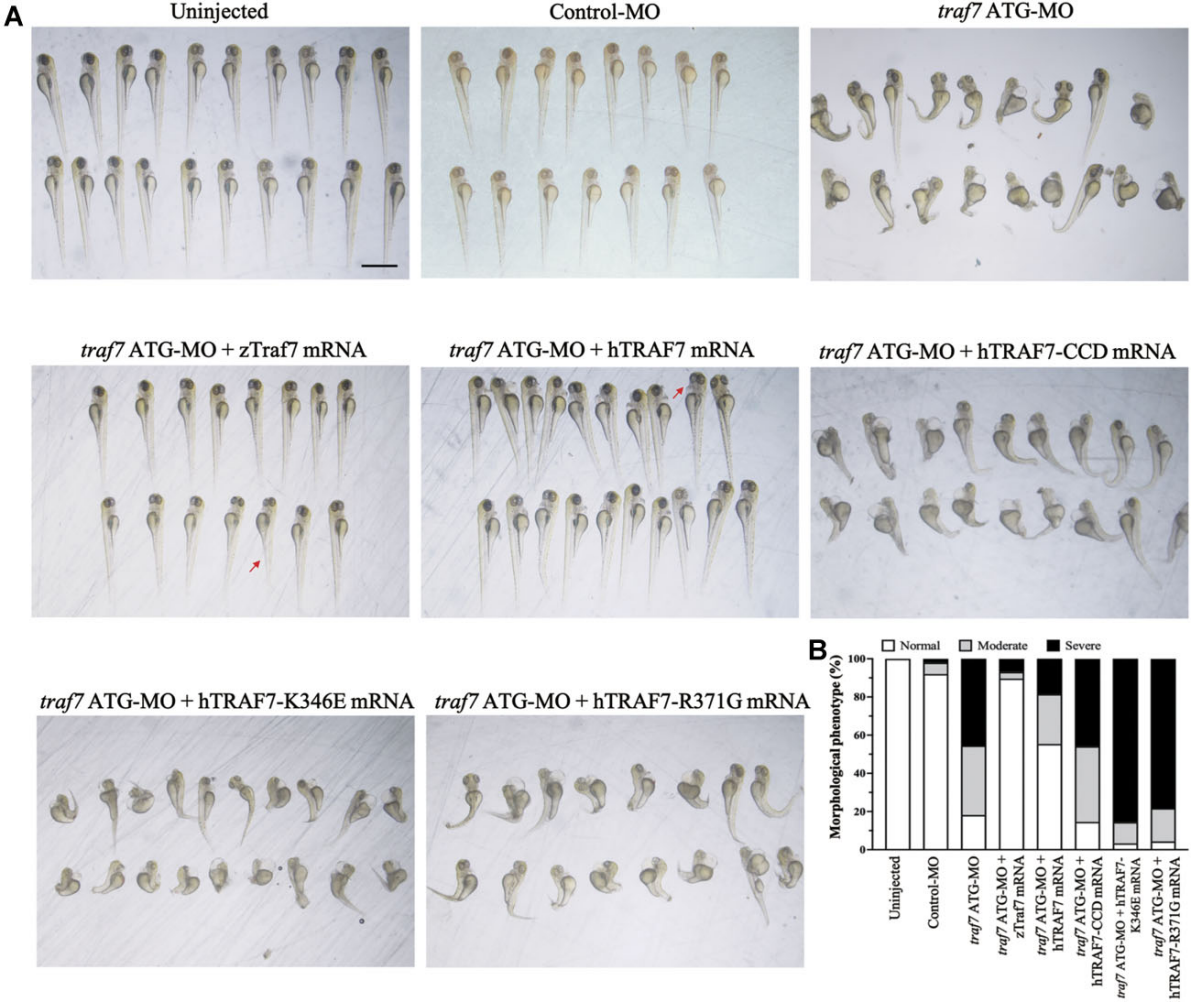
CC region mutant TRAF7 fails to rescue developmental defects in *traf7* ATG-MO-injected zebrafish embryos. (**A**) Rescue using zebrafish wild-type Traf7 (zTraf7), human wild-type TRAF7 (hTRAF7), and human TRAF7 CC region mutants (hTRAF7-CCD, hTRAF7-K346E, and hTRAF7-R371G). Red arrows indicate malformation features. Scale bar, 1 mm. (**B**) Morphological phenotype of living embryos after rescue at 3 dpf.

Furthermore, we conducted rescue experiments by reintroducing zebrafish Traf7 (zTraf7), human TRAF7 (hTRAF7), human CC-deleted TRAF7 (hTRAF7-CCD), human K346E TRAF7 (hTRAF7-K346E), or human R371G TRAF7 (hTRAF7-R371G) mRNA (Supplementary Figure S5) back into the embryos in 1-cell or 2-cell stage. Each mRNA was co-injected with *traf7* ATG-MO at a concentration of 100 pg. As shown in Figure 6, the abnormal phenotypes due to Traf7 knockdown were largely rescued by wild-type zTraf7 or hTRAF7 mRNA but not by hTRAF7-CCD, hTRAF7-K346E, or hTRAF7-R371G mRNA. These results confirmed that disruption of TRAF7 CC region (hTRAF7-CCD) or weakening the trimer formation (hTRAF7-K346E) affects TRAF7 function in zebrafish embryonic development. Therefore, our study underscores the essential role of TRAF7 CC domain trimerization in zebrafish embryonic development and offers valuable insights into TRAF7-associated pathogenesis.

## Discussion

Zebrafish is a widely used animal model in developmental biology (Fulwiler and Gilbert, 1991), including research on TRAF families. Given the potential for gene editing and phenotype observation, it offers a promising model to analyze TRAF7 function and associated signaling pathways in early development, with sequences highly conserved from human to zebrafish. In 2004, the spatial and temporal distribution of the *traf4* genes was reported during zebrafish development (Kedinger et al., 2005). Another experiment provided a structural model of the RING and ZF domains of zebrafish TRAF6 and affirmed its comparable function to human TRAF6 (Middleton et al., 2017). In a recent study investigating the effects of TRAF7 mutations, researchers found that mutations in TRAF7 caused congenital heart defects in zebrafish (Mishra-Gorur et al., 2023).

In our study, we detailed the spatial and temporal expression patterns of the *traf7* gene during zebrafish development, revealing prominent expression in the head region. Knockdown of Traf7 caused developmental defects in zebrafish, partially consistent with the clinical manifestations found in patients with *TRAF7* pathogenic variants. Patients with TRAF7 mutations frequently present short stature, skull shape anomalies, and axial skeleton anomalies (Tokita et al., 2018; Castilla-Vallmanya et al., 2020).

**Figure 7:**
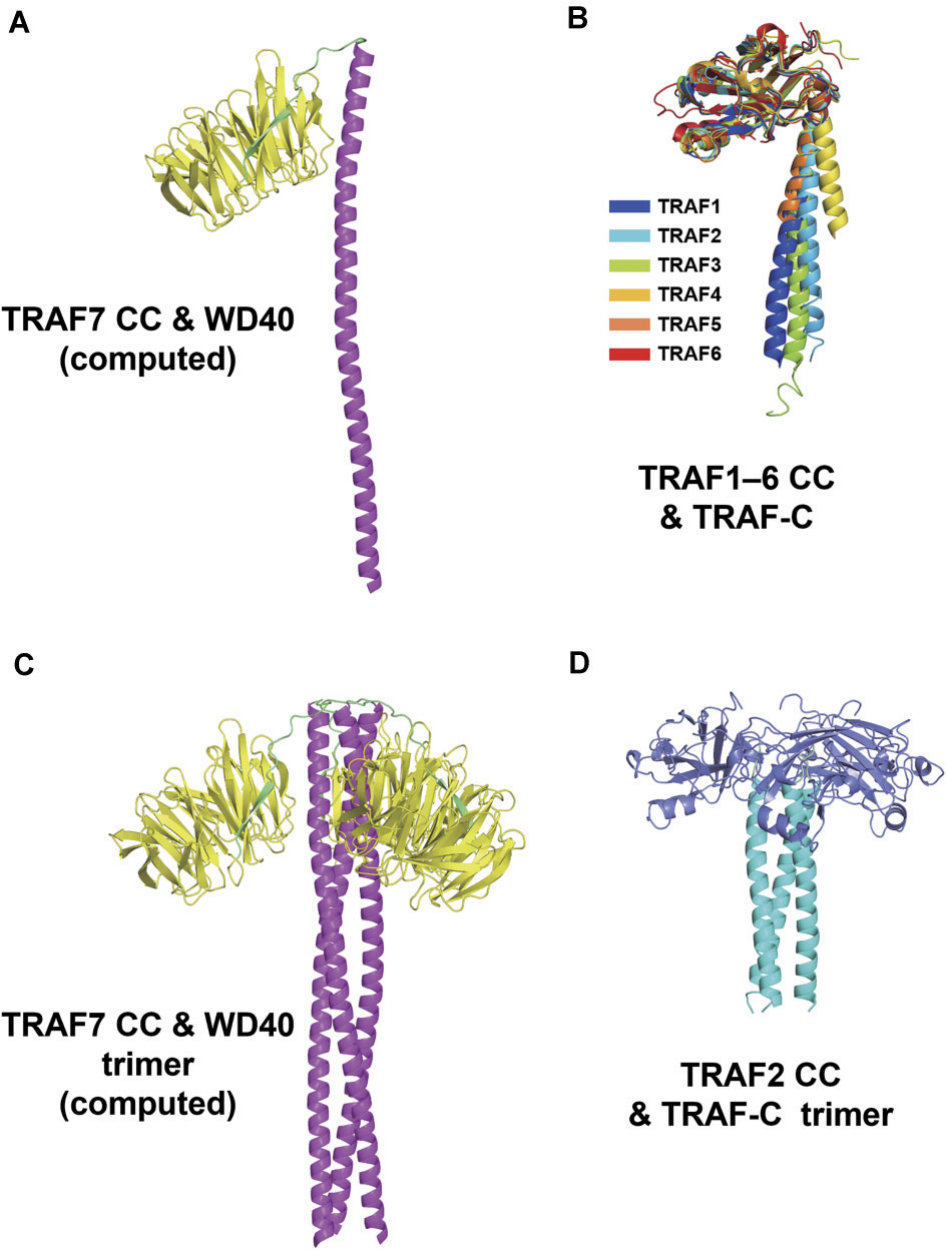
C-terminal structural alignment of TRAF family members. (**A**) AlphaFold-predicted structure of TRAF7 CC and WD40 domains. The CC region (288–374 aa) is a long α-helix colored magenta, while WD40 (385–670 aa) is colored yellow. (**B**) Alignment of crystal structures of TRAF1–6 CC and TRAF-C regions. TRAF1–4 and TRAF6 are from humans, and TRAF5 is from mice. TRAF1–5 are homotrimers in crystals, which only show monomers. PDB ID: TRAF1, 5E1T; TRAF2, 1CA9; TRAF3, 1FLK; TRAF4, 3ZJB; TRAF5, 4GJH; TRAF6, 1LB4. (**C**) TRAF7 CC and WD40 trimer generated by AlphaFold2.3.1 with TRAF7 homotrimer input. (**D**) TRAF2 CC and TRAF-C trimer from the crystal structure (PDB ID: 1CA9). The CC domain (311–347 aa) is an α-helix colored cyan. The TRAF-C domain (352–501 aa) is colored slate blue.

TRAF proteins form a trimeric, mushroom-like structure in solution via their CC domain to perform critical biological functions (Rothe et al., 1994; Park et al., 1999; Niu et al., 2013; Kim et al., 2016), and TRAF1–6 TRAF-C domains also have similar structures. However, TRAF7 is an exception because seven WD40 repeats replace the TRAF-C domain at the C-terminus, as depicted in the AlphaFold computed structure (Supplementary Figure S6). Focusing on the C-terminus of TRAF7, including the CC region and WD40 domain, we found that the computed structure of TRAF7 C-terminus was similar to that of TRAF1–6 CC and TRAF-C domains (Figure 7A and B). TRAF7 CC and WD40 domains were proven to mediate several TRAF7 functions. The CC domain interacts with NEMO (Zotti et al., 2011), while the WD40 domain interacts with MEKK3 and c-myb (Xu et al., 2004; Morita et al., 2005). Experiments involving pathogenic variants showed that TRAF7-K346E had no significant effect on the phosphorylation of ERK1/2, while TRAF7-R371G decreased ERK1/2 phosphorylation (Tokita et al., 2018). Combining these findings with our structural results, it is reasonable to suppose that K346E undermines the stability of TRAF7 itself, whereas R371G impairs the interaction between TRAF7 and potential binders related to the MAPK pathway. The structural analysis comparing the computed TRAF7 trimer (Figure 7C) and TRAF2 trimer (Figure 7D) demonstrated the long and flexible link between TRAF7 CC and WD40 domains, suggesting a specific function.

Our *in vivo* experiments illustrated the importance of TRAF7 CC trimer to its function. In a cohort of seven *TRAF7* syndrome patients, Tokita et al. (2018) reported two *TRAF7* variants (K346E and R371G) in the CC domain, demonstrating the potential of these mutations to cause severe developmental disease in humans. Our zebrafish rescue experiments showed that zebrafish or human wild-type TRAF7 mRNA effectively rescued malformed phenotypes, while three mutant TRAF7 mRNAs carrying CC region mutations could not. A cohort of 45 individuals harboring *TRAF7* missense variants identified four variants (D327E, D363E, S366F, and A370V), further underscoring the pathogenicity of CC region variants (Castilla-Vallmanya et al., 2020).

In conclusion, our results showed that TRAF7 CC region formed a stable functional trimer in solution, and the formation and stabilization of the CC trimer are crucial for zebrafish embryonic development. Our current structure study elucidated the CC region structures of the entire TRAF family.

## Materials and methods

### Zebrafish maintenance

The wild-type TU strain zebrafish and the transgenic line Tg(*huC*:*RFP*) were obtained from Shanghai Ruijin Hospital. In all of the zebrafish experiments, zebrafish embryos were cultured in ‘egg water’ that included 0.002% methylene blue and 0.03% sea salt as a fungicide. Zebrafish were kept in a circulating water system of 27°C–28°C with a 14-h light/10-h dark cycle. Water from all tanks was circulated into every tank after passing through a 120-μm filter pad, a 5-μm filter cartridge, an activated carbon filter, a biological filter, and a UV disinfection filter. To maintain the water quality in the circulatory system, pH, general hardness, carbonate hardness, nitrite, and nitrate were monitored weekly. Daily feeding included artificial feed pellets or live brine shrimp.

### WISH analysis of gene expression

A 515 bp cDNA fragment of *traf7* was subcloned and inserted into the pClone007 simple vector (Tsingke), which was conserved across species, with the following primers: F: 5′-CCTCCATTGCTCACTATGAA-3′; R: 5′-AGTAGATCGCCTGTCGAATA-3′. The digoxigenin-labeled sense and antisense probes were synthesized using Sp6 and T7 mMESSAGE mMACHINE kits (Ambion). Wild-type TU zebrafish embryos were collected and fixed with 4% paraformaldehyde in phosphate-buffered saline (PBS) overnight at 4°C. Then, the embryos were dehydrated with a mixture of gradient methanol and PBST (Tween-20, 1‰) and stored in 100% methanol at –20°C. WISH was performed as previously described (Topczewski et al., 2001).

### Morpholinos, mRNA microinjection, and TUNEL assay

MOs were purchased from Gene Tools. The morpholino sequences are shown in Supplementary Table S2. Capped mRNA for rescue was transcribed from linearized PCS2+ plasmids (mMessage Machine; Ambion), purified, and diluted to 50–100 ng/μl for microinjection at the 1-cell to 2-cell stages (Supplementary Table S1). After 72 h, the embryos were observed with microscopy, the phenotypes were scored, and photographs were taken using a QImaging microscope system. The TUNEL assay was performed according to the kit instructions (YF 488 TUNEL Apoptosis Kit, UElandy).

### Protein expression and purification

Human TRAF7 (GenBank: AY569455.1) CC fragments (270–379 aa) and ZF-CC fragments (221–379 aa) were cloned and inserted into pET32M3C to produce TRAF7 proteins with a TrxA tag and His tag at the N-terminus. Various mutations were generated using standard PCR-based methods and confirmed by sequencing. The TRAF7 constructs were transformed into *Escherichia coli* BL21 (DE3) cells and cultured in LB medium at 37°C. When the OD600 of the culture was 0.6, IPTG (0.2 mM) was added to induce protein expression, and the cells were cultured at 16°C for 16 h. The proteins were purified using a nickel-NTA agarose affinity column followed by size-exclusion chromatography with a Superdex 200 column. Tags were cleaved by HRV 3C protease at 4°C overnight when needed and separated by another process of size-exclusion chromatography. The protein storage buffer contained 50 mM Tris (pH 8.0), 100 mM NaCl, 10 mM EDTA, and 1 mM DTT.

### Crystallization and structure determination

TRAF7 CC crystals were obtained by the sitting drop vapor diffusion method at 16°C. The protein was crystallized by mixing 1 μl of fresh purified protein (5 mg/ml in 50 mM Tris, pH 8.0, 100 mM NaCl, 10 mM EDTA, and 1 mM DTT) with 1 μl crystallization buffer containing 0.1 M HEPES (pH 6.9) and 0.2 M magnesium formate dihydrate.

All diffraction data were collected at the BL19U beamlines of Shanghai Synchrotron Radiation Facility (SSRF) and processed with HKL2000 (https://www.hkl-xray.com/). The structure of TRAF7 CC was solved by molecular replacement using the structure of TRAF2 CC domain (PDB ID: 3M06) as the search model. Model building was performed in the program PHENIX (https://www.phenix-online.org/). Coot (http://www2.mrc-lmb.cam.ac.uk/personal/pemsley/coot/) was used for model refinement. All structure figures were generated using the program PyMOL (http://pymol.sourceforge.net/).

### MALS analyses

The protein samples of TRAF7 CC, TRAF7 ZF-CC, and their mutants were loaded into a Superdex 200 gel filtration column in storage buffer (50 mM Tris, pH 8.0, 100 mM NaCl, 10 mM EDTA, and 1 mM DTT). The chromatography system was linked to a three-angle light scattering detector (mini-DAWN TRISTAR) and a refractive index detector (Optilab DSP) (Wyatt Technology). ASTRA V (https://www.wyatt.com/products/software/astra.html) was used for data analysis.

### Data Availability

The atomic coordinate of TRAF7 CC trimer is deposited in the Protein Data Bank under the accession code 8IMS. Other datasets for this study are included in the article (or Supplementary material).

## Supplementary Material

mjad083_Supplemental_File

## Notes

**Author contributions:** Q.L. and S.W. designed and supervised the study; X.S. and R.H. performed the main experiments; Y.C. and M.X. performed partial *in vivo* experiments; X.S., R.H., and Y.C. drafted the manuscript; H.Z. provided critical feedback. All authors discussed the final results and critically reviewed the manuscript. All authors have read and agreed to the published version of the manuscript.
