# Supplementary material for: The structure of TRAF7 coiled-coil trimer provides insight into its function in zebrafish embryonic development
Source: J Mol Cell Biol. 2024 Jan 4;16(1):mjad083. doi: 10.1093/jmcb/mjad083 (PMC11216086; doi:10.1093/jmcb/mjad083)
Supplement: mjad083_Supplemental_File [file mjad083_supplemental_file.pdf]

Supplementary material

The structure of TRAF7 coiled-coil trimer provides insight into its function in zebrafish embryonic development

Xiaozhen Song[1,†], Ruixing Hu[2,†], Yi Chen[3,†], Man Xiao[1], Hong Zhang[1], Shengnan Wu[1,*], and Qing Lu[2,*]

[1] Molecular Diagnostic Laboratory, Shanghai Children's Hospital, School of Medicine, Shanghai Jiao Tong University, Shanghai 200040, China

[2] Bio-X Institutes, Key Laboratory for the Genetics of Developmental and Neuropsychiatric Disorders (Ministry of Education), Shanghai Jiao Tong University, Shanghai 200030, China

[3] Laboratory of Development and Diseases and State Key Laboratory for Medical Genomics, Shanghai Institute of Hematology, Ruijin Hospital, Shanghai Jiao Tong University School of Medicine, Shanghai 200025, China

[†] These authors contributed equally to this work.

* Correspondence to: Shengnan Wu, E-mail: wushengnan@shchildren.com.cn; Qing Lu, E-mail: luqing67@sjtu.edu.cn

Supplementary Tables

Supplementary Table S1 Primer sequences used in zebrafish experiments.

	**Forward primer**	**Reverse primer**
RT-PCR test	CAGCAGTCCCACAAGTAGCA	GCAGAATGTGTGTCCACAGG
pCS2+ zebrafish traf7-res	TAGCGAATTCGCCGCCACCatggatgctggtttt ggatc	TAGCCTCGAGttagcacgtccacacctttac
pCS2+ human TRAF7-res	tagcGAATTCgccaccatgagctcaggcaagagtgcccgc	tagCTCGAGttagcaagtccaaaccttcacagtgc
pCS2+ human K346E TRAF7-res	F1: gacgaaaaccagagcgagctcagcgaggacct<br>F2: TACAAGCTACTTGTTCTTTTTGCAGGA TCCgccaccatgagctcaggcaagagtgcc	R1: ggtcctcgctgagctcgctctggttttcgtcc<br>R2: TTCTAGAGGCTCGAGAGGCCTTGAAT TCttagcaagtccaaaccttcacagt
pCS2+ human R371G TRAF7-res	F1: tcccacatcaacgcggggctgaacatgggcat<br>F2: TACAAGCTACTTGTTCTTTTTGCAGGA TCCgccaccatgagctcaggcaagagtgcc	R1: tgcccatgttcagccccgcgttgatgtgggac<br>R2: TTCTAGAGGCTCGAGAGGCCTTGAAT TCttagcaagtccaaaccttcacagt
pCS2+ human CCD TRAF7- res	F1: tacgggtgcacgttcatctacgaccctcagcagatcttcaag<br>F2: GCAGGATCCCATCGATTCGAATTCgcc accatgagctcaggcaagagtgcccgc	R1: gtcgtagatgaacgtgcacccgtacttgga<br>R2: TCACTATAGTTCTAGAGGCTCGAGttag caagtccaaaccttcacagt
pClone007-traf7-WISH	CCTCCATTGCTCACTATGAA	AGTAGATCGCCTGTCGAATA

Supplementary Table S2 Sequences of morpholino injected in zebrafish larvae.

**Morpholino**	**Sequence**
traf7 ATG-MO	CATTTTGGCCTTGTTGGTGATGTCC
traf7 E3i3-MO	TGGTCCAACAGTTAATACTGACCTT
traf7 E4i4-MO	CAGAAGATTGCTGACACTCACCGTG
traf7 5MIS-MO	CATTaTcGCgTTGTTGcTcATGTCC

Supplementary Figures

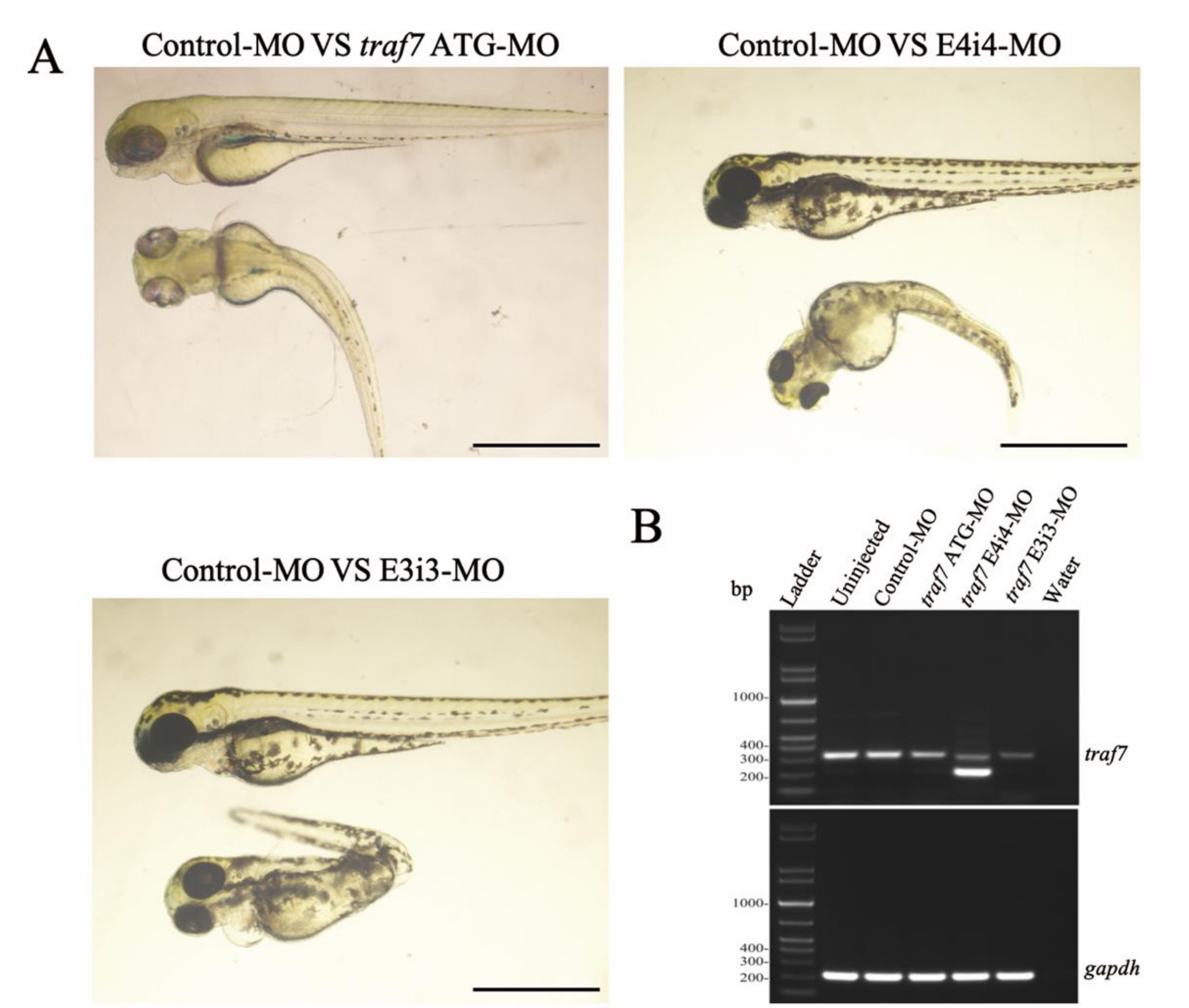

Supplementary Figure S1 Loss of Traf7 in zebrafish induced abnormal embryonic development. (**A**) Compared with control-MO group, zebrafish showed similar abnormal phenotypes after injection with t*raf7* ATG-MO, Splicing-blocking MO *traf7* E3i3-MO or *traf7* E4i4-MO at 3 dpf. Scale bar, 1mm. (**B**) RT-PCR for *traf7* expression in *traf7* E3i3-MO and *traf7* E4i4-MO injected zebrafish showed *traf7* expression effectively reduced compared to uninjected and Control-MO groups.

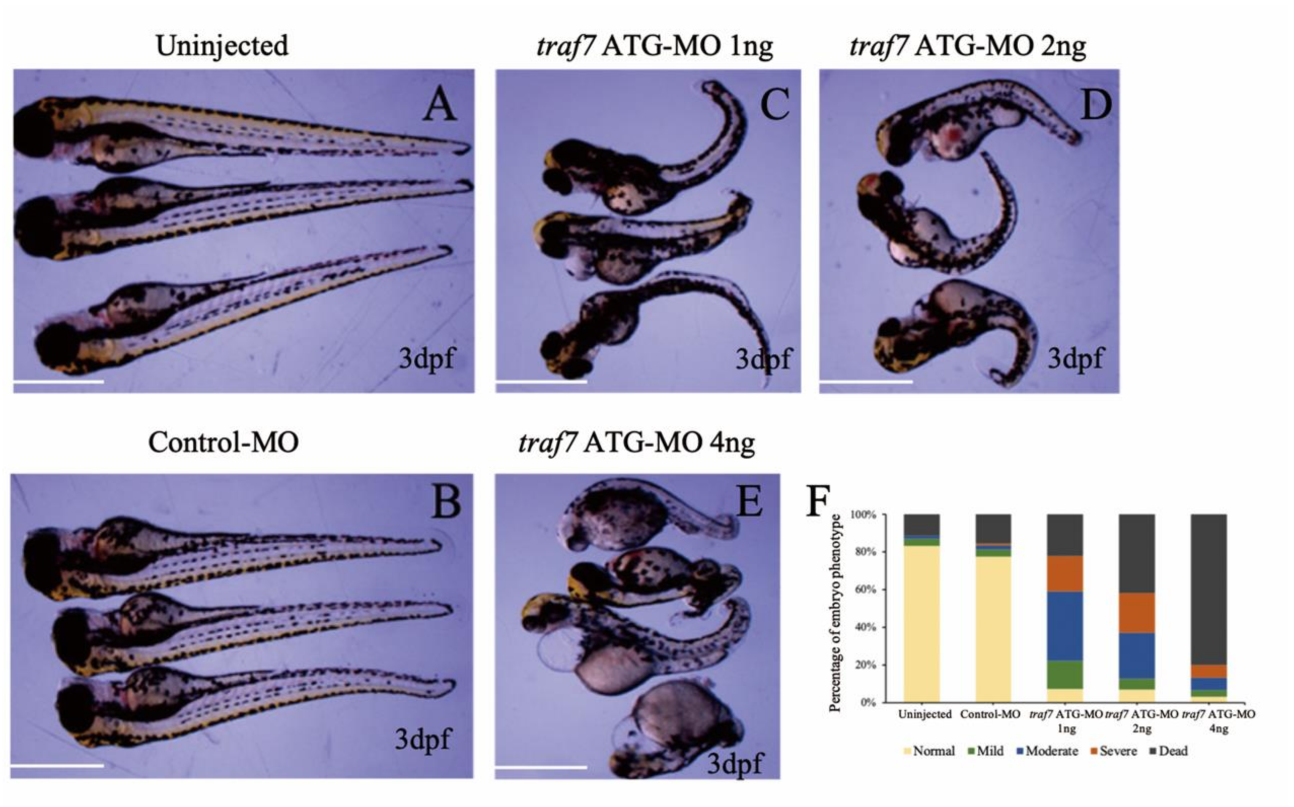

Supplementary Figure S2 Morphology of *traf7* knockdown zebrafish based on *traf7 ATG-MO* at 3 dpf. (**A–E**) The severity of morphology was dose dependent after injection of *traf7 ATG-MO*. Scale bar, 1 mm. (**F**) The phenotypic classification of morphant.

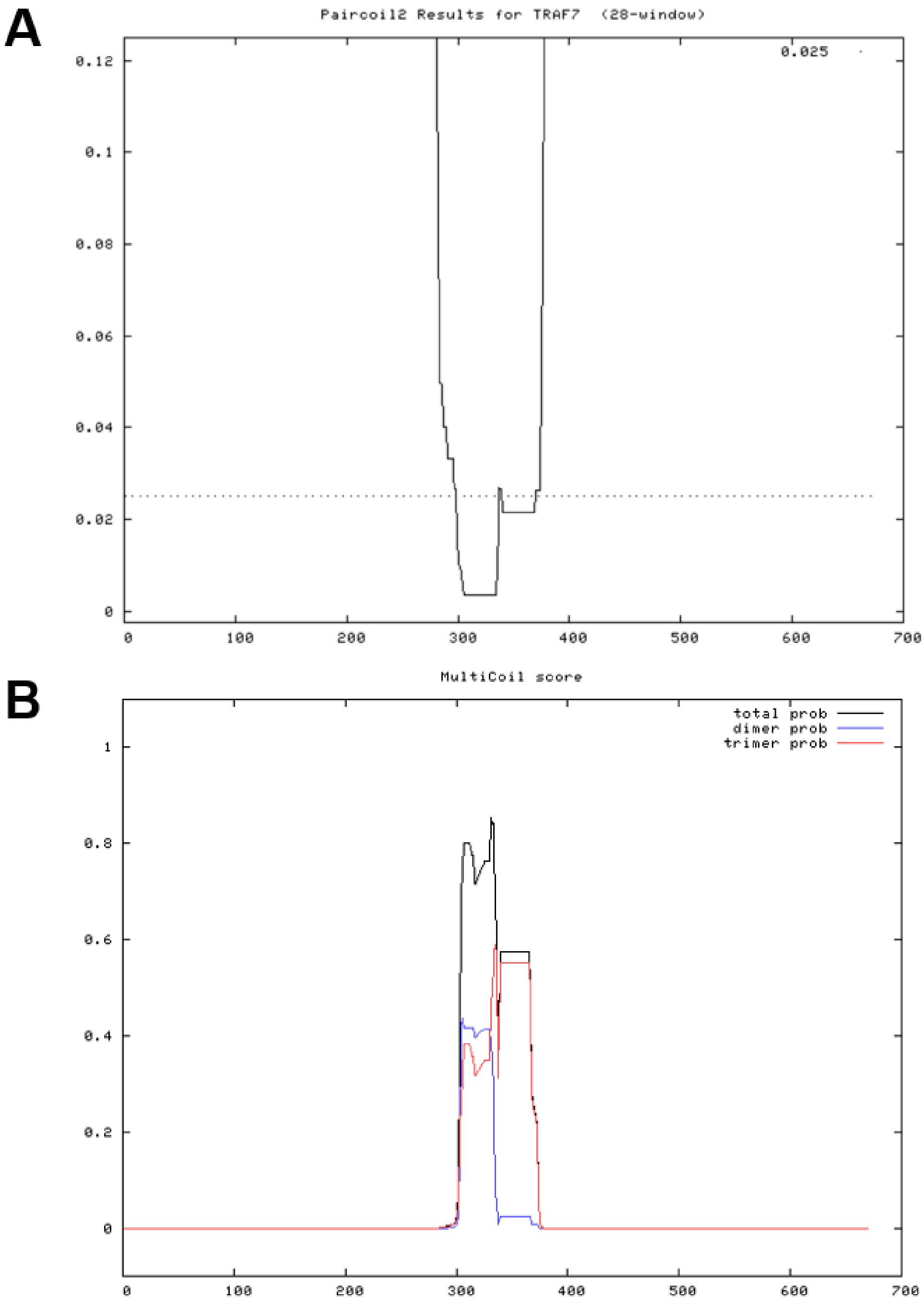

Supplementary Figure S3 Prediction of TRAF7 CC region by Paircoil2 and MultiCoil.

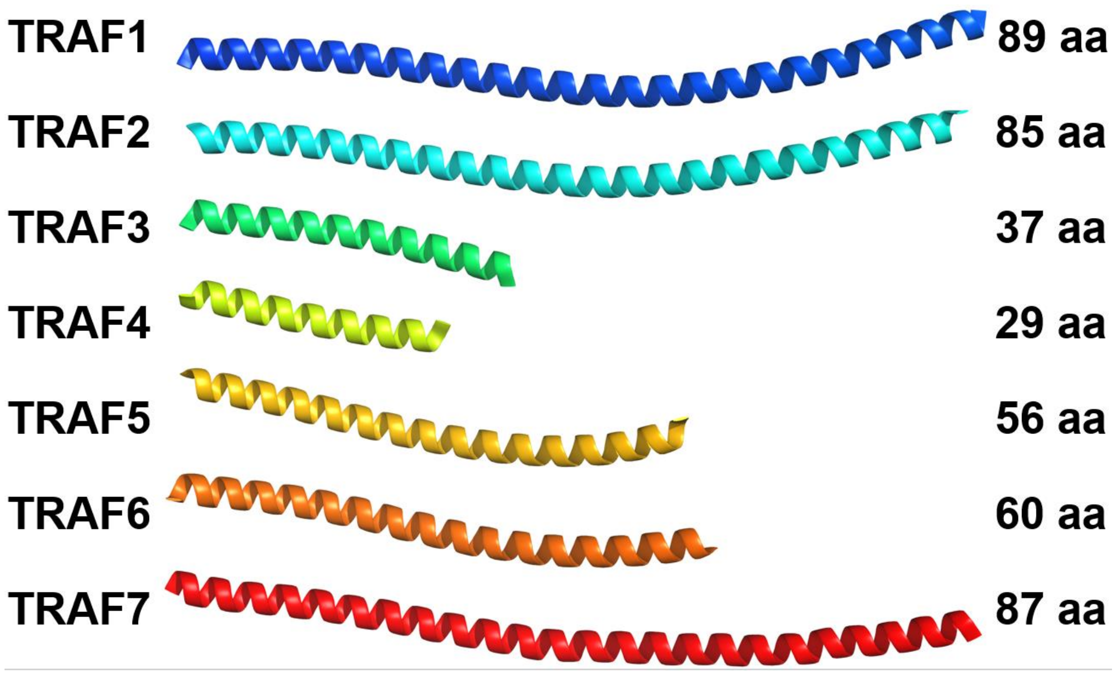

Supplementary Figure S4 Structure overlay of CC domain among paralogs, based on AlphaFold predicted structure. Compared to TRAF1–6, TRAF7 CC (87 aa of each chain) contains many residues similar to TRAF1/2, and in experimental structure TRAF7 CC (91 aa of each chain) is longer than TRAF1-6.

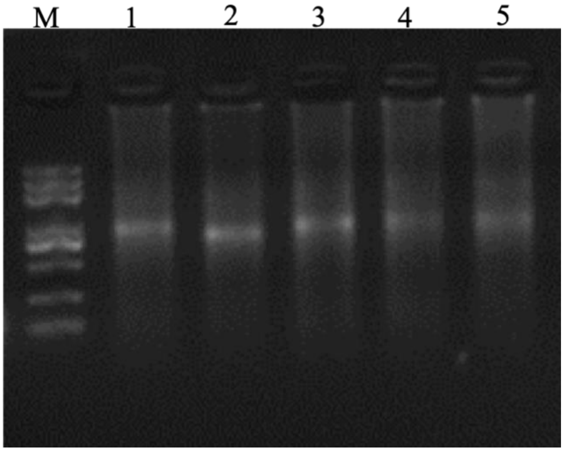

Supplementary Figure S5 Agarose gel electrophoresis identification results of purified mRNA. The first lane M is DNA Marker: 100 bp, 250 bp, 500 bp, 750 bp, 1000 bp, 1500 bp, 2000 bp, 3000 bp, 5000 bp from bottom to top; Lanes named 1–5 were purified hTRAF7-WT, hTRAF7-CCD, hTRAF7-R371G, zTraf7-WT and hTRAF7-K346E mRNA, respectively.

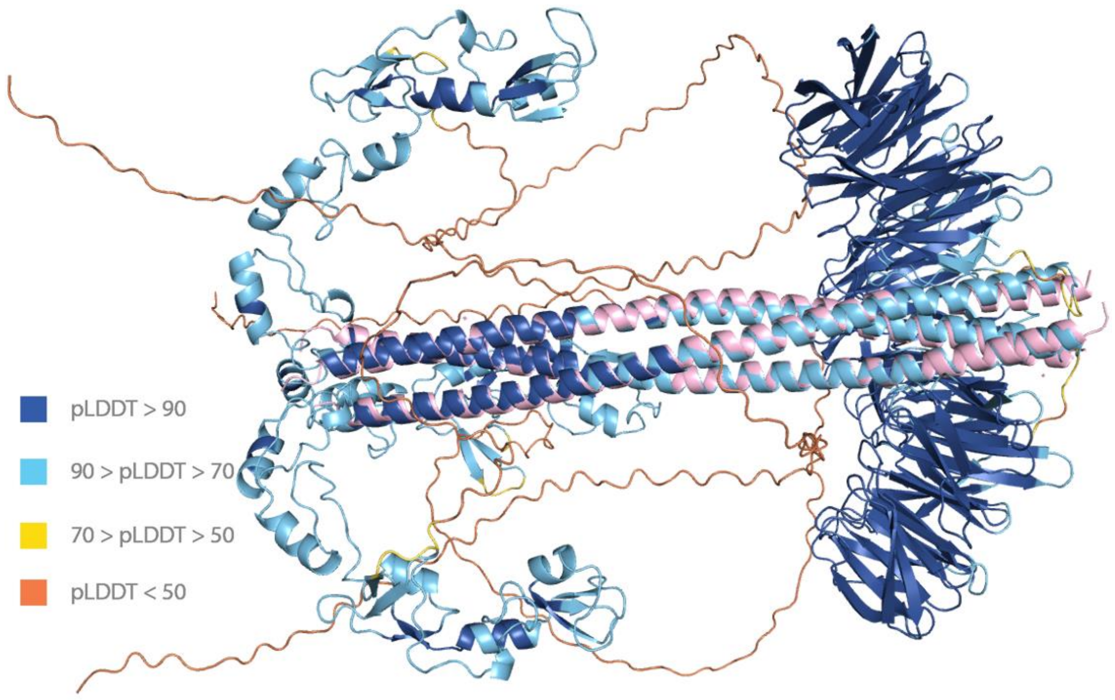

Supplementary Figure S6 Experimental structure of TRAF7 trimer (colored light pink) aligned with AlphaFold2.3.1 computed structure.
